# Differences in soil bacterial community structure during the remediation of Cd-polluted cotton fields by biochar and biofertilizer in Xinjiang, China

**DOI:** 10.3389/fmicb.2024.1288526

**Published:** 2024-02-09

**Authors:** Yongqi Zhu, Mengjie An, Tumur Anwar, Haijiang Wang

**Affiliations:** ^1^Key Laboratory of Biological Resources and Genetic Engineering of Xinjiang Uygur Autonomous Region, College of Life Science and Technology, Xinjiang University, Ürümqi, Xinjiang, China; ^2^Agricultural College, Shihezi University, Shihezi, Xinjiang, China

**Keywords:** soil heavy metal contamination, bacterial diversity, cadmium enrichment, bioremediation, cotton

## Abstract

**Introduction:**

Heavy metal pollution is a major worldwide environmental problem. Many remediation techniques have been developed, these techniques have different performance in different environments.

**Methods:**

In this study, soil sampling was conducted in multiple cotton fields in Xinjiang, China, and found that cadmium (Cd) was the most abundant soil heavy metal. Then, to find the most suitable technique for the remediation of Cd pollution in cotton fields, a two-year study was conducted to explore the effects of cotton straw-derived biochar (BC, 3%) and *Bacillus*-based biofertilizer (BF, 1.5%) on cotton Cd uptake and transport and soil microbial community structure under Cd exposure conditions (soil Cd contents: 1, 2, and 4 mg·kg^−1^).

**Results:**

The results showed that the bioaccumulation coefficients (Cd content of cotton organs / soil available Cd content) of cotton roots, stems, leaves, and buds/bolls reduced by 15.93%, 14.41%, 23.53%, and 20.68%, respectively after the application of BC, and reduced by 16.83%, 17.15%, 22.21%, and 26.25%, respectively after the application of BF, compared with the control (no BC and BF). Besides, the application of BC and BF reduced the transport of Cd from soil to root system, and enhanced the diversity of soil bacterial communities (dominant species: *Alphaproteobacteria* and *Actinobacteria*) and the metabolic functions related to amino acid synthesis. It was worth noting that the differential species for BF group *vs* BC group including *Alphaproteobacteria*, *Gemmatimonadetes*, *Bacilli*, and *Vicinamibacteria* were associated with the enrichment and transport of Cd, especially the transport of Cd from cotton roots to stems.

**Discussion:**

Therefore, the application of BC and BF changed the soil bacterial diversity in Cd-polluted cotton field, and then promoted the transport of Cd in cotton, ultimately improving soil quality. This study will provide a reference for the selection of soil heavy metal pollution remediation techniques in Xinjiang, China.

## 1 Introduction

Soil heavy metal pollution remediation is a hot topic worldwide. In 2018, China issued the *Standard for Risk Control of Arable Soil Pollution* (GB 15618-2018), a standard for the evaluation of heavy metal pollution in arable soil in China. Studies have shown that the content of heavy metal in arable soil in Xinjiang gradually increases (Zheng et al., 2018; Abudureheman et al., 2021). For example, Ma et al. (2021) reported that the contents of Cr, Cu, Zn, As, and Cd in the arable soil in Altay, Xinjiang exceeded the average values of Xinjiang, and the average Cd content was as high as 0.20 mg·kg^−1^, which seriously affected crop growth and threatened human health. Zheng et al. (2018) reported that the contents of Zn, As, Pb, Cr, and Cu were very high in the soil of Bole, Changji, and Kashgar in Xinjiang.

Soil microorganisms play a very important role in soil biogeochemical cycles and ecosystem functions, and have been widely used to assess soil health (Song J. W. et al., 2022). Under heavy metal pollution, soil microbial ribosomes, RNA polymerase, DNA polymerase, RNA and protein processing, and carbon sequestration are inhibited to varying degrees. Besides, the higher the content of heavy metals, the more obvious the inhibition is (Ma et al., 2021). The genetic basis of microbial resistance to heavy metals includes energy-dependent efflux (ATPase, RND, CDF family), enzymatic detoxification (redox and demobilization), and cell fixation and uptake (Senthil Kumar et al., 2023). Besides, soil bacterial communities such as *Acinetobacter*, *Citrobacter*, and *Pseudomonas* resist heavy metal stresses through enzymatic detoxification or transforming heavy metals into nontoxic forms by intracellular/extracellular binding (Wu et al., 2021). Therefore, the study of soil microbial diversity, structure, and function is of great significance for the remediation of heavy metal pollution.

Biochar produced by high-temperature anaerobic pyrolysis of manure, plant straw, wood, etc. has been widely used to reduce the bioavailability of soil heavy metals (Lin et al., 2022; Wan et al., 2022). The rich pore structure and large specific surface area of biochar provide a good habitat for soil microorganisms, and its rich nutrients such as organic matter, nitrogen, and phosphorus stimulate soil microbial growth and metabolism (Liu et al., 2021). Studies have shown that biochar increases not only the relative abundance of microorganisms associated with the carbon-nitrogen cycle (Actinobacteria, and *Pseudomonas*), but also increases the bioavailability of chemically bonded phosphates in soil. Besides, it also plays a great role in the fixation of free metal ions (Ahmad et al., 2017). Some components in biochar, such as water-soluble nutrients, affect soil microbial activity, and increase soil nutrient content and organic carbon mineralization, which ultimately promotes bacterial growth and activity and increases soil bacterial community diversity (*Proteobacteria*, *Bacteroidetes*, *Gemmatimonadetes*, *Actinobacteria*, *Nitrospirae*, and *Patescibacteria*; Wang B. H. et al., 2020; Sun T. et al., 2023). Biofertilizer, as another material for the remediation of soil heavy metal pollution, can increase the diversity of beneficial bacteria in the soil, and these beneficial bacteria can promote the growth and development of plants and the adsorption of soil pollutants such as heavy metals. In addition, the application of biofertilizer can also promote the secretion of metabolites of plants, and reduce the morbidity of plants, ultimately increasing soil quality, crop yield and quality (Wei et al., 2023). Recent studies have shown that inoculating the soil with *Bacillus* can not only increase crop yield and resistance to external abiotic stresses, but also reduce the bioavailability of inorganic and organic pollutants such as heavy metals (Han et al., 2018), plant diseases (Sun Y. et al., 2023), and polycyclic aromatic hydrocarbons (Song L. et al., 2022) in the soil. Dong et al. (2019) also found that the application of *Bacillus*-rich biofertilizer significantly increased the total saponin content of *Panax notoginseng* roots by 51.49%, and promoted the shoot and root biomass accumulation. It can be seen that the application of biofertilizer with *Bacillus* as the dominant bacterium not only has a positive effect on crop growth and soil quality, but also reduces the risk of environmental pollution and the transportation and accumulation of pollutants in the food chain (Wang et al., 2019).

It can be seen that the use of biochar and biofertilizer in the remediation of heavy metal pollution in arable soil is feasible. Biochar and biofertilizer can not only reduce the content of available Cd in the soil, but also improve the living environment of soil microorganisms and soil quality (soil pH, nutrients, and physical characteristics; Zhu Y. et al., 2022). Our previous study results showed that biochar and biofertilizer had different effects on soil available Cd content, soil physicochemical properties, and cotton Cd uptake (Zhu Y. Q. et al., 2022). To further explore the reasons behind this from the perspective of soil microorganisms, in this study, the effects of cotton straw-derived biochar (BC, 3%) and *Bacillus*-based biofertilizer (BF, 1.5%) on cotton Cd migration and transformation and soil microbial community structure were investigated under Cd exposure conditions (soil Cd contents: 1, 2, and 4 mg·kg^−1^). This study hypothesized that the application of BC and BF might reduce the bioavailability and migration of soil Cd by adjusting soil bacterial community structure. The objectives of this study were to clarify: (1) the most abundant heavy metal in the cotton fields in Xinjiang, (2) the effects of BC and BF application on the content of Cd in cotton organs and the quality of Cd-polluted soil (soil available Cd content, soil bacterial community and diversity), and (3) the reasons for the difference in the effects of BC and BF on soil bacterial community structure based on the function prediction of dominant species. This study will provide a reference for the selection of soil heavy metal pollution remediation technologies and the improvement of arable soil quality in arid areas.

## 2 Materials and methods

### 2.1 Study site

Xinjiang is located in northwest China (34°25′–49°10′N, 73°40′–96°23′E), with an arid climate. The average annual precipitation was 150 mm, and the average annual temperature was 33°C. The favorable climate and advanced cotton planting technology have made Xinjiang the largest cotton production base in China. According to statistics, Xinjiang’s cotton planting area accounted for 60%–70% of the total arable land in Xinjiang, and most cotton fields had been continuously cropped for 10–15 years. In this study, soil sampling was carried out in eight regions of Xinjiang, including Changji, Shihezi, Bole, Kuitun, Shawan, Korla, Aksu, and Kashgar, according to the ranking of cotton planting area and cotton yield (Zheng et al., 2018).

#### 2.1.1 Soil sampling

Continuous cotton cropping is very common in Xinjiang. In this study, 60 cotton fields (>33.3 × 103 m^2^) that had been continuously cropped for more than 10 years were selected (Supplementary Figure S1) for soil sampling (0~20 cm soil layer) from September to October 2020. In each cotton field, five points were selected along the diagonals, and three soil samples were collected from each point. The soil samples of a cotton field were mixed and divided into three equal parts. Finally, a total of 180 soil samples were collected (1 kg per sample). After removing impurities such as stones and plant roots, the soil samples were brought back to the laboratory to be air-dried and sieved for the determination of soil heavy metal content.

#### 2.1.2 Determination of soil physical and chemical properties and soil heavy metal content

The contents of As, Cd, Cr, Cu, Ni, and Pb in the soil samples were determined by graphite furnace atomic absorption spectrophotometer (Z2000, Hitachi, Tokyo, Japan), after digesting the samples in concentrated nitric acid, concentrated hydrochloric acid, and hydrofluoric acid (Zhu Y. et al., 2022).

The soil heavy metal survey results showed that soil Cd content exceeded the background value the most. To test the impacts of Cd, CdCl_2_·5H_2_O was mixed with soil to prepare soils with different Cd concentrations (1 (H1), 2 (H2), 4 (H3) mg·kg^−1^), and then outdoor pot (40 cm in height, 25 cm in diameter) experiment was conducted at the Experimental Station of Agricultural College, Shihezi University, Xinjiang (44°18′42.37”N, 86°03′20.72″E). H1, H2, and H3 were about 4, 8, and 16 times the average soil Cd content in Xinjiang. After 60 days, the following tests were carried out.

#### 2.1.3 Materials

The preparation method of BC was as follows: Cotton straw was crushed in a muffle furnace under hypoxia condition at 450°C for 6 h. After cooling to room temperature in the muffle furnace, the straw were ground and sieved through a 0.15 mm sieve. The conversion rate from cotton straw to BC was 37.5%. The BF used in this study was purchased, and the physicochemical properties were determined according to the national standard of China (GB 20287-2006; Supplementary Table S1).

### 2.2 Experimental design

This experiment had 12 groups totally (Table 1), and each treatment had five replicates. Fifteen cotton seeds (variety Xinluzao 53, a widely cultivated variety in Xinjiang) were sown in a ceramic pot in April 2019/2020, and 5–6 plants were retained when the true leaves were fully unfolded. A total of 345 kg·hm^−2^ of urea (N), 555 kg·hm^−2^ of compound fertilizer (N-P_5_O_2_-K_2_O, 17–17-17), and 4.8 kg·hm^−2^ of potassium polyacrylate (K_2_O) were applied during the whole growth period. All phosphorus and potassium fertilizers and half of the urea were applied before sowing, and the rest urea was applied after the bud stage. After 120 days of culture, cotton organs (roots, stems, leaves, and buds/bolls) were collected, dried in an oven at 105°C, to determine dry matter yield and Cd content. At the same time, soil samples were collected from the pots using wooden shovel. The samples of each group were mixed. About 100 g was used for the determination of soil Cd content and available Cd content after air-drying and sieving, and the rest was used for bacterial diversity analysis. The sampling methods and tests were consistent in 2019 and 2020.

**Table 1:** Experimental design.

Treatments	Cd (mg·kg^−1^)	Biochar (%)	Biofertilizer (%)
H0T (Control)	0.25	0	0
H0B	0.25	3%	0
H0J	0.25	0	1.5%
H1T	1	0	0
H1B	1	3%	0
H1J	1	0	1.5%
H2T	2	0	0
H2B	2	3%	0
H2J	2	0	1.5%
H3T	4	0	0
H3B	4	3%	0
H3J	4	0	1.5%

T, no modifiers; B, 3% biochar was applied; J, 1.5% biofertilizer was applied; H0, no Cd; H1, 1 mg·kg^−1^ of Cd was applied; H2, 2 mg·kg^−1^ of Cd was applied; H3, 4 mg·kg^−1^ of Cd was applied.

The translocation factor (TF) and bioaccumulation coefficient (BCF) of Cd were used to characterize the migration and uptake of Cd in cotton, respectively (Amin et al., 2023).

$$TF=\frac{\mathrm{Root}/\mathrm{Stem}/\mathrm{Leaf}/\mathrm{Boll}\;Cd\;\mathrm{content}}{\mathrm{Soil}/\mathrm{Root}/\mathrm{Stem}/\mathrm{Leave}\;Cd\;\mathrm{content}}$$

$$\mathrm{Bioaccumulation coefficient}\;\left(BCF\right)=\frac{Cd\;\mathrm{content in}\;a\;\mathrm{plant organ}}{Cd\;\mathrm{content in soil}}$$

The available Cd content in soil and the Cd content in cotton organs are shown in Supplementary Tables S2, S3 (Yu et al., 2022).

### 2.3 Parameters and measurement methods

#### 2.3.1 Determination of Cd content in cotton organs

The available Cd in the Cd-polluted soils was extracted by diethylenetriaminopentaacetic acid (DTPA; Zheng et al., 2018). To determine the Cd content in cotton roots, stems, leaves, and buds/bolls, 0.5 g of dried sample of each cotton organ was digested with the mixture of nitric acid and perchloric acid (2:1, v/v) under sealed condition, followed by the determination with a graphite furnace atomic absorption spectrophotometer (Z2000, Hitachi, Tokyo, Japan; Yang et al., 2016).

#### 2.3.2 Determination of soil bacterial community diversity

Based on the 16S rRNA gene, high throughput sequencing was performed to determine soil bacterial diversity. The DNA extraction procedure was as follows: phosphoric acid buffer (pH: 8.0) and Tris(hydroxymethyl)methyl aminomethane (pH: 8.0) were mixed with soil sample (0.5 g). Then, the mixture was broken using a disruptor (Fastprep-24, United States), and centrifuged to obtain the supernatant. After that, the sample was extracted with reagents PCI (phenol: chloroform: isoamyl alcohol = 25: 24: 1) and CI (chloroform: isoamyl alcohol = 24: 1), followed by a centrifugation. The obtained precipitate was dissolved in Tris-EDTA buffer solution (pH: 8.0) to obtain the DNA solution.

The DNA solution was purified and sent to Shanghai Paysenno Co., Ltd. for high-throughput sequencing. The paired-end sequencing of bacterial DNA fragments was carried out on the illumina Mi Seq 300PE platform, and the obtained sequences were subjected to de-priming, filtering, denoising, splicing, and dechimerism using the DA-DA2 method. The sequences obtained were the representative sequences of operational taxonomic units (OTUs). Subsequently, sequence analysis was conducted using the QIIME2 software package, specifically employing the qiime dada2 denoise-single, qiime feature-table summarize, and qiime feature-table tabulate-seqs tools. Finally, the diversity indices (Chao1, Coverage, Simpson, and Shannon indices) of soil bacteria were calculated by IIME 2 software (Puga et al., 2015; Kiran and Prasad, 2019). Fisher’s exact test was carried out to detect species with abundance difference between groups, and hypothesis testing was carried out to evaluate the significance of observed differences (Romanowski et al., 2023). Bacterial diversity analysis was performed under the soil Cd content 4 mg·kg^−1^ (H0T, H3T, H3B, and H3J).

### 2.4 Data analysis

Before data analysis, normal distribution was tested using the Kolmogorov–Smirnov (K-S) test using SPSS 20.0 software (SPSS Inc., Chicago, United States). Data were expressed as mean ± standard error. Duncan test was performed to test the significance of differences in bioaccumulation coefficients and translocation factor between groups using SPSS 20.0 software (SPSS Inc., Chicago, United States; *p* < 0.05). The maximum, minimum, average, standard error, median, and variability of each element content in the soil samples were statistically analyzed using Excel software version 2016 and SPSS software version 23.0. Figures were drawn using Origin software version 8.0 (Origin Lab, Massachusetts, United States), and layout was completed using Adobe Illustrator CS6 (Adobe, United States).

## 3 Results

### 3.1 The contents of soil heavy metals in cotton fields in Xinjiang

The Cd content in soil was twice the background value in Xinjiang. Among the heavy metals, Cd had the highest coefficient of variation (43.72%), followed by Pb (42.34%), Cr (38.95%), Ni (35.65%), Cu (35.05%), and As (31.27%; Table 2).

**Table 2:** Descriptive statistics of soil heavy metal content in cotton filed in Xinjiang, China (mg·kg^−1^).

Heavy metal	As	Cd	Cr	Cu	Ni	Pb
Statistics analysis						
Mean	6.64	0.24	37.60	19.24	13.80	15.20
Coefficient of variation	0.31	0.44	0.39	0.35	0.36	0.42
Minimum	1.34	0.01	11.77	8.95	3.46	5.99
Maximum	12.38	0.47	92.39	78.67	27.73	33.36
Standard deviation	2.09	0.10	14.64	12.45	4.92	6.42
Median	6.65	0.25	36.45	15.35	13.49	14.34
Soil background values in Xinjiang	11.20	0.12	49.30	26.70	26.60	19.40
Threshold values in the *Standard for Risk Control of Arable Soil Pollution* (GB 15618-2018)	25	0.60	250	100	60	170
Percentage of soil samples with heavy metal content exceeding the background value	3.33%	88.33%	23.33%	15.00%	1.67%	25.00%

### 3.2 The translocation factor and bioaccumulation coefficient of Cd

**Figure 1:**
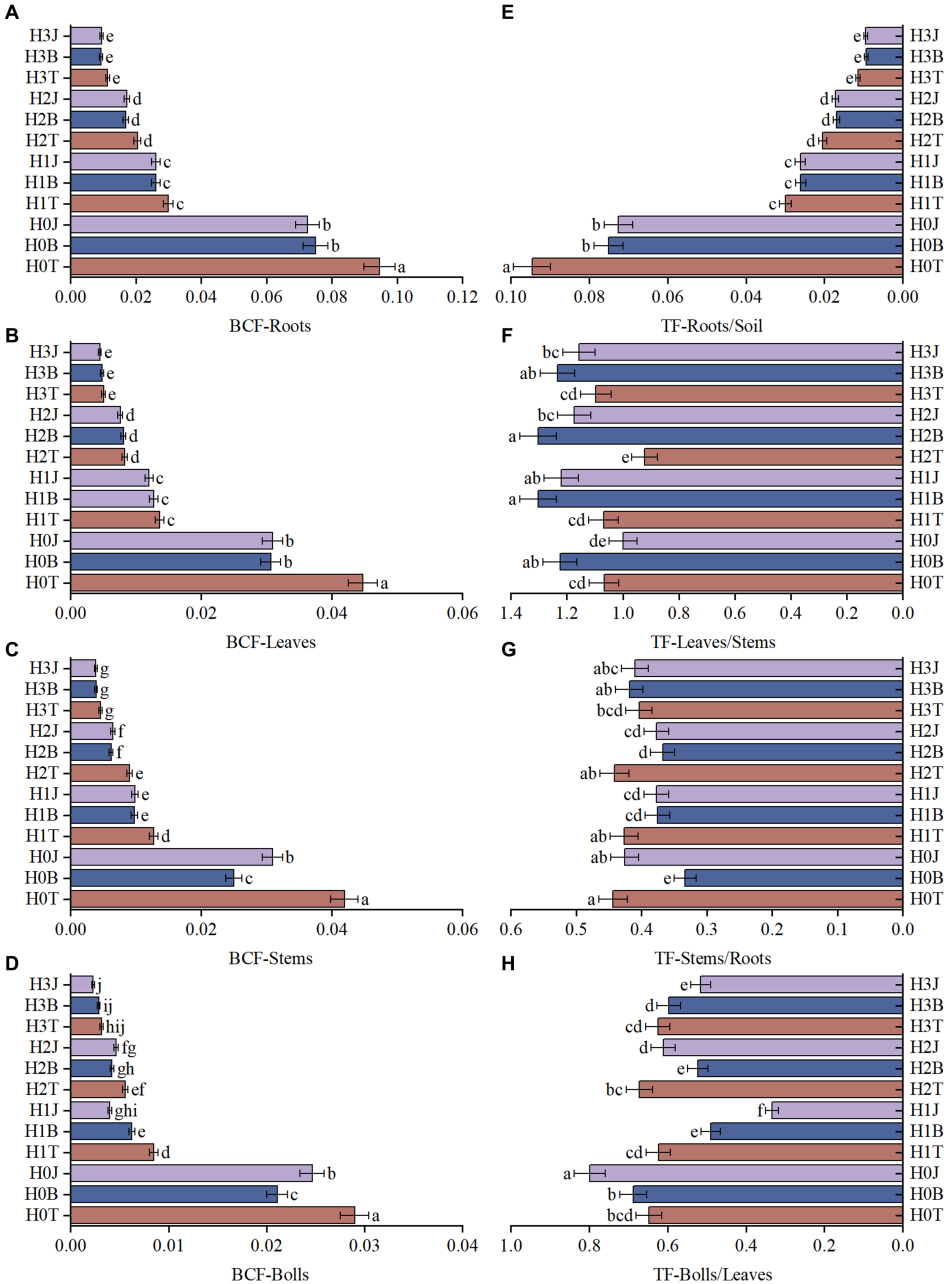
Bioaccumulation coefcients (BCFs) and Translocation factor (TF) of Cd in cotton organs (2019). (A) BCF-Roots; (B) BCF-Leaves; (C) BCF-Stems; (D) BCF-Bolls; (E) TF-Roots/Soil; (F) TF-Leaves/Stems; (G) TF-Stems/Roots; (H) TF-Bolls/Leaves. Diferent lowercase letters indicae signifcant diference between groups at *p* < 0.05. T, no modifiers; B, 3% biochar was applied; J, 1.5% biofertilizer was applied; H0, no Cd; H1, 1 mg·kg^−1^ of Cd was applied; H2, 2 mg·kg^−1^ of Cd was applied; H3, 4 mg·kg^−1^ of Cd was applied. The same below.

During the two-year culture period, with the increase of exogenous Cd concentration, the Cd content in cotton roots, stems, leaves, and buds also increased. For example, the content of Cd in cotton roots in the H1T, H2T, and H3T groups increased by 11.01% (*p* > 0.05), 29.46% (*p* < 0.05), and 45.24% (*p* < 0.05), respectively compared with that in the H0T group. The BC treatment reduced the Cd content in cotton organs. For example, the content of Cd in cotton roots in the H0B, H1B, H2B, and H3B groups decreased by 20.54% (*p* < 0.05), 12.87% (*p* > 0.05), 17.47% (*p* > 0.05), and 18.24% (*p* < 0.05), respectively compared with the H0T, H1T, H2T, and H3T group. The BF treatment also reduced the Cd content in various organs of cotton. For example, the Cd content in cotton roots in the H0J, H1J, H2J, and H3J groups decreased by 23.81% (*p* < 0.05), 12.60% (*p* > 0.05), 15.86% (*p* > 0.05), and 17.01% (*p* < 0.05), respectively compared with the H0T, H1T, H2T, and H3T group (Supplementary Tables S2, S3). The BCF of Cd of cotton roots was higher than that in other organs in all groups, but the BC and BF treatments reduced the BCF of each organ. In 2019 (2020), the BCF of roots, leaves, stems, and buds/bolls in the H0B group reduced by 20.64% (14.28%), 31.53% (30.39%), 40.34% (10.51%), and 27.28% (25.09%), respectively (*p* < 0.05) compared with those in the H0T group (Figures 1A–D). The BCF of roots, leaves, stems, and buds/bolls in the H0J group reduced by 23.27% (14.72%), 30.96% (29.81%), 26.33% (17.61%), and 14.94% (26.27%), respectively (*p* < 0.05), compared with those in the H0T group (Supplementary Figures S2A,B).

The BC and BF treatments reduced the transport of soil Cd to cotton roots. In 2019 and 2020, the TF-Roots/Soil in the H0B group reduced by 20.64 and 14.28%, respectively (*p* < 0.05), and the TF-Roots/Soil in the H0J group reduced by 23.27 and 14.71%, respectively (*p* < 0.05), compared with that in the H0T group. However, the BC and BF treatments increased the transport of stem Cd to cotton leaves. In 2019 and 2020, the TF-Leaves/Stems in the H1B group reduced by 21.89 and 14.19%, respectively (*p* < 0.05; Figures 1E–H), and the TF-Leaves/Stems in the H1J group reduced by 14.06 and 14.32%, respectively (*p* < 0.05), compared with that in the H1T group (Supplementary Figures S2E–H).

### 3.3 Soil bacterial α-diversity and β-diversity

**Figure 2:**
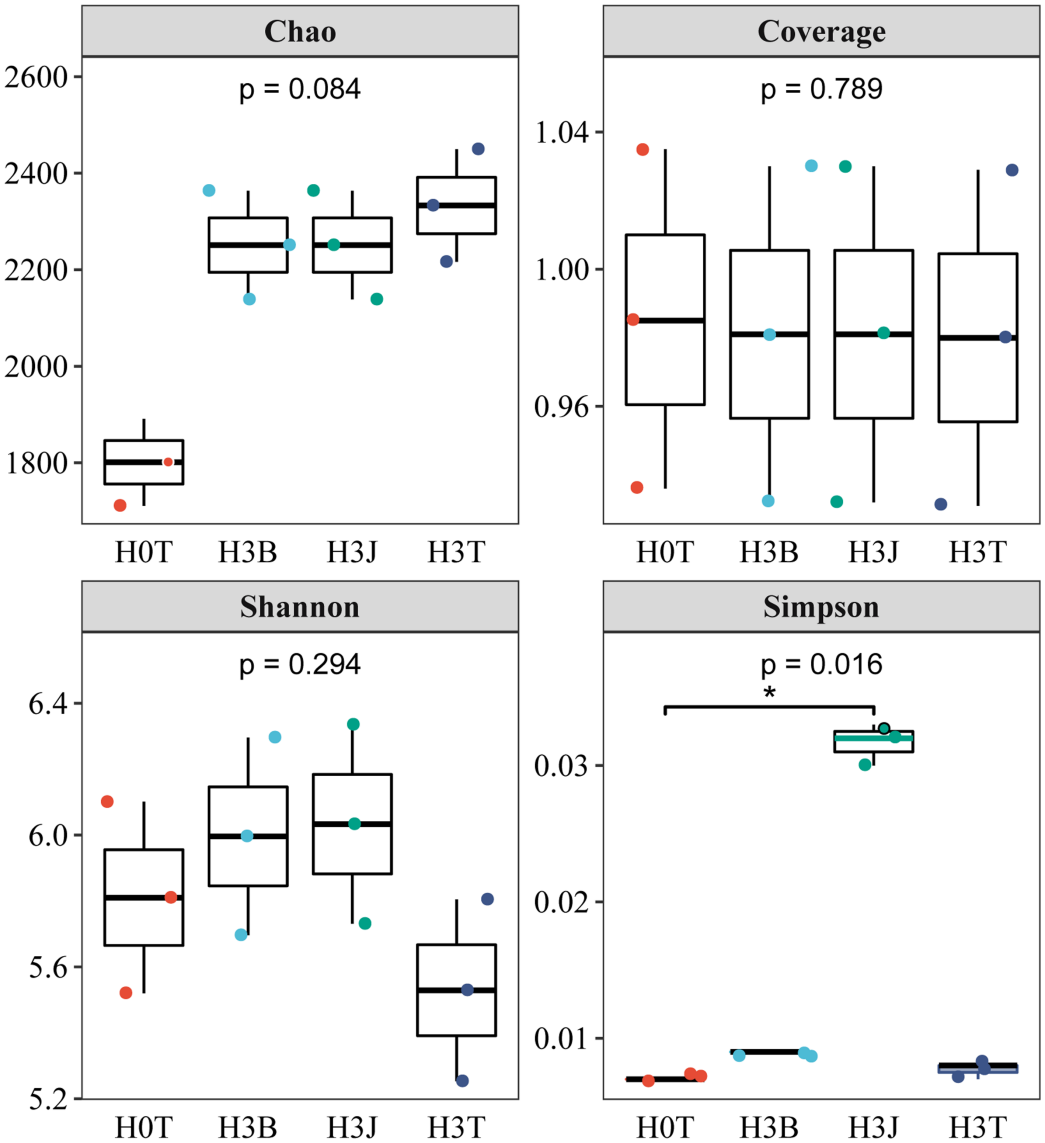
Effect of biochar (B) and biofertilizer (J) on microbial α-diversity in Cd contaminated soil (2019).

In 2019 (Figure 2), the Shannon index in the H3T group reduced by 4.84% compared with that in the H0T group (*p* > 0.05). The analysis of coverage index showed that the sequencing coverage of each sample was above 97.97%, reflecting the reliability of the sequencing results. The Simpson’s diversity index in the H3B group increased by 15.38% (*p* < 0.05), the Chao 1 index reduced by 3.71% (*p* < 0.05), and the Shannon index increased by 8.44% (*p* > 0.05), compared with those in the H3T group. The Simpson’s diversity index in the H3J group increased by 303.9% (*p* < 0.05), the Chao 1 index reduced by 3.73% (*p* < 0.05), and the Shannon index increased by 50.41% (*p* > 0.05), compared with those in the H3T group.

**Figure 3:**
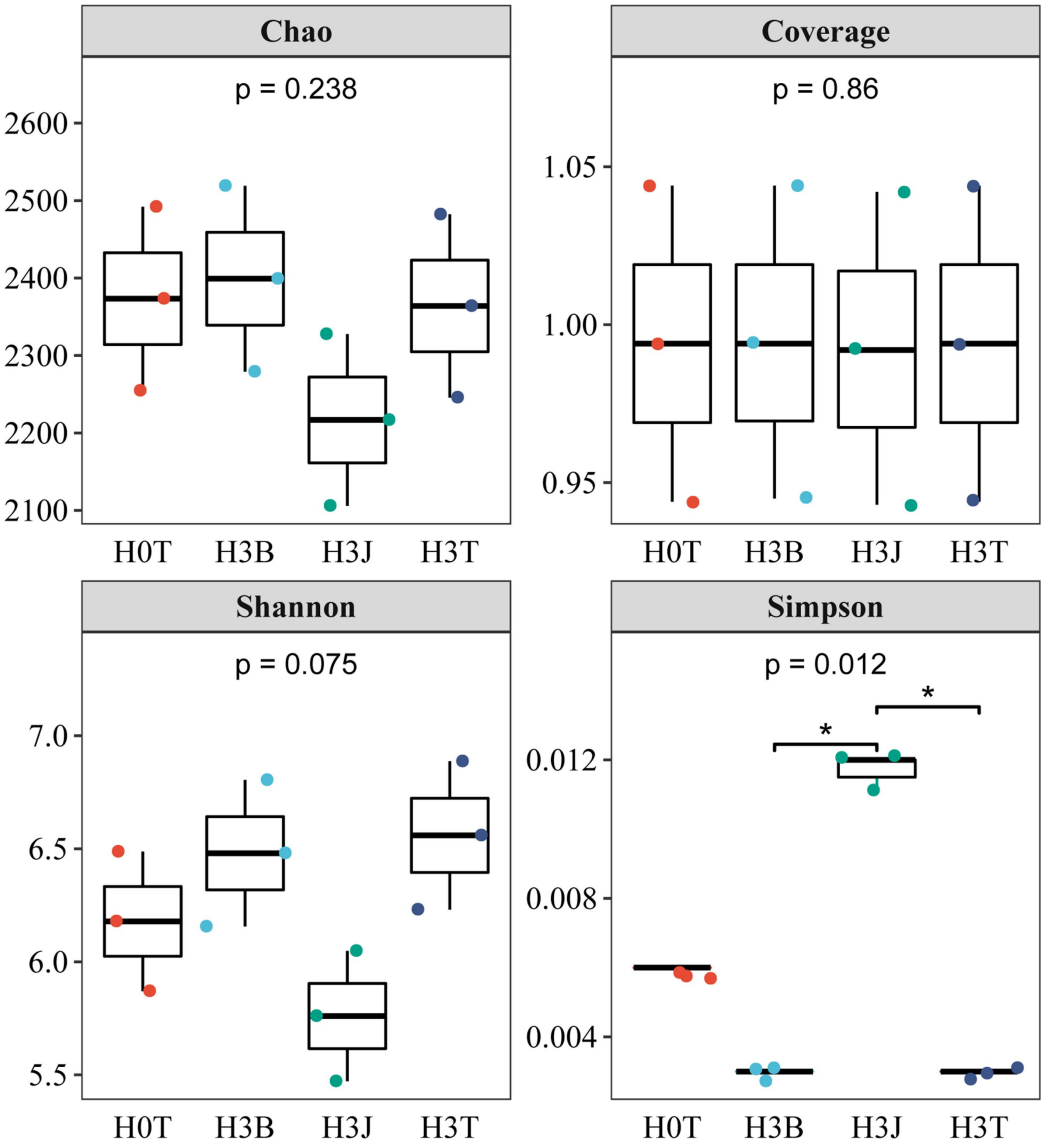
Effects of biochar (B) and biofertilizer (J) on microbial α-diversity in Cd contaminated soil (2020).

In 2020 (Figure 3), there was no significant difference in the Chao 1 and coverage indices between groups. The Simpson index in the H3T group reduced by 50.91% (*p* < 0.05) compared with that in the H0T group. The Shannon index in the H3B group reduced by 12.18%, and the Simpson index in the H3B and H3J group increased by 298.84% and 734.52%, respectively (*p* < 0.05), compared with those in the H3T group.

The soil microbial β-diversity was similar in 2019 and 2020 (Supplementary Figure S3). The distribution of the β-diversity in the H3B and H3J groups was close in Supplementary Figures S3A,B, indicating that the effects of BC and BF treatments on soil microbial β-diversity were similar. However, the distribution of the soil microbial β-diversity in the H0T and H3T groups were far away, indicating that the addition of Cd greatly affected the soil bacterial community structure.

### 3.4 Changes in relative abundance of soil bacteria at the class level

**Figure 4:**
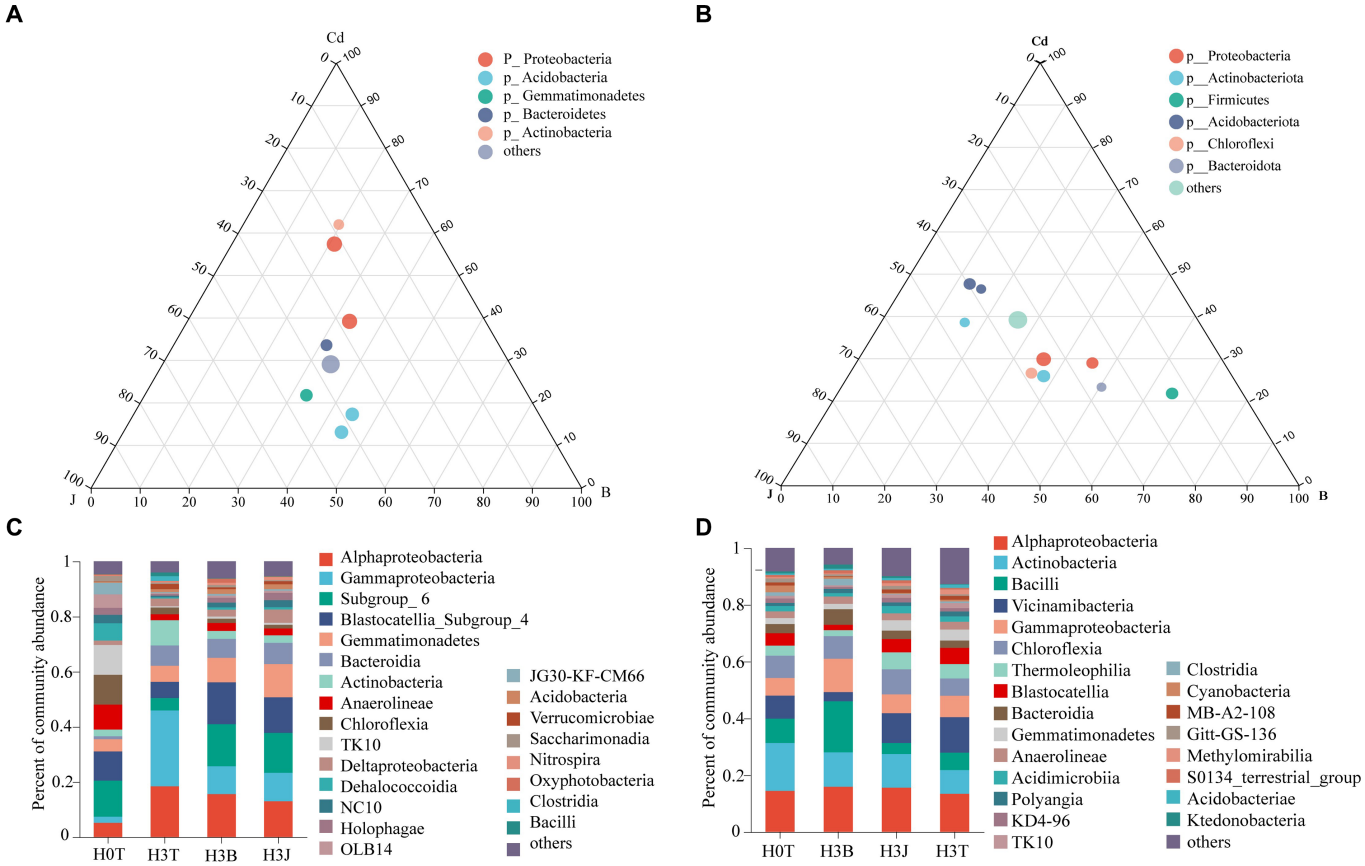
Efects of biochar [B] and biofertilizer [J] on bacterial diversity in Cd contaminated soil (2019 and 2020). **(A)** Ternary phase diagram of 2019; **(B)** Ternary phase diagram of 2020; **(C)** Percent of community abundance in 2019; **(D)** Percent of community abundance in 2020.

The Cd, BC, and BF treatments had significant effects on the relative abundance of soil bacteria in 2019 and 2020. In 2019, *Alphaproteobacteria* (5.14%–18.41%), *Gammaproteobacteria* (2.24–27.50%), *Subgroup_6* (4.48%–15.25%), *Blastocatellia_Subgroup_4* (5.90%–15.23%), *Gemmatimonadetes* (4.44%–12.07%), *Bacteroidia* (1.08%–7.70%), *Actinobacteria* (2.46%–9.13%), *Anaerolineae* (2.16%–8.99%), and *Chloroflexia* (1.33%–10.81%) were the dominant bacteria in the groups. The ternary phase diagram showed that the soil bacterial diversity varied among different samples (Figures 4A,B). *Actinobacteria* (9.13%) and *Gemmatimonadetes* (5.86%) were the dominant bacteria in the H3T group, and *Blastocatellia_Subgroup_4* (15.23% and 12.94%) and *Gemmatimonadetes* (8.88 and 12.07%) were the dominant bacteria in the H3B and H3J groups (Figure 4A). Besides, it was found that the relative abundance of *Blastocatellia_Subgroup_4* and *Gemmatimonadetes* in the H3B group increased by 9.33 and 3.03%, respectively, while that of *Alphaproteobacteria* and *Gammaproteobacteria* reduced by 2.83% and 17.42%, respectively, compared with those in the H3T group. The relative abundance of *Blastocatellia_ Subgroup_ 4* and *Gemmatimonadetes* in the H3J group increased by 7.04% and 6.22%, respectively, while that of *Alphaproteobacteria* and *Gammaproteobacteria* decreased by 5.44% and 17.18%, respectively, compared with those in the H3T group. The relative abundance of *Gemmatimonadetes* in the H3J group increased by 3.19% compared with that in the H3B group (Figure 4C).

In 2020, *Alphaproteobacteria* (13.42%–15.93%), *Actinobacteria* (8.36%–16.88%), *Bacilli* (3.92%–17.97%), *Vicinamibacteria* (3.26%–12.54%), *Gammaproteobacteria* (6.18%–11.73%), *Chloroflexia* (6.08%–8.80%), *Thermoleophilia* (2.11%–5.98%), *Blastocatellia* (1.86%–5.70%), and *Bacteroidia* (2.57%–5.51%) were the dominant bacteria in the groups. The ternary phase diagram (Figure 4B) showed that *Vicinamibacteria*, *Thermoleophilia*, and *Bacilli* were the dominant bacteria in the H3T, H3J, and H3B group, respectively. The relative abundance of *Alphaproteobacteria*, *Actinobacteria*, and *Bacilli* in the H3B group increased by 2.51%, 3.74%, and 11.85%, respectively, while that of *Vicinamibacteria* decreased by 9.27%, compared with those in the H3T group. The relative abundance of *Alphaproteobacteria*, *Actinobacteria*, and *Chloroflexia* in the H3J group increased by 2.14%, 3.49%, and 2.72%, respectively, while that of *Bacilli* and *Gammaproteobacteria* decreased by 2.19% and 0.86%, respectively, compared with those in the H3T group. Besides, the relative abundance of *Vicinamibacteria* in the H3J group increased by 7.21% compared with that in the H3B group (Figure 4D).

In 2019, *Alphaproteobacteria* and *Gemmatimonadetes* were the differential bacteria for the H3J group *vs* H3B group (Supplementary Figure S4A). In 2020, *Bacilli* and *Vicinamibacteria* were the differential bacteria for the H3J group *vs* H3B group (Supplementary Figure S4B).

### 3.5 Prediction of soil bacterial functions

**Figure 5:**
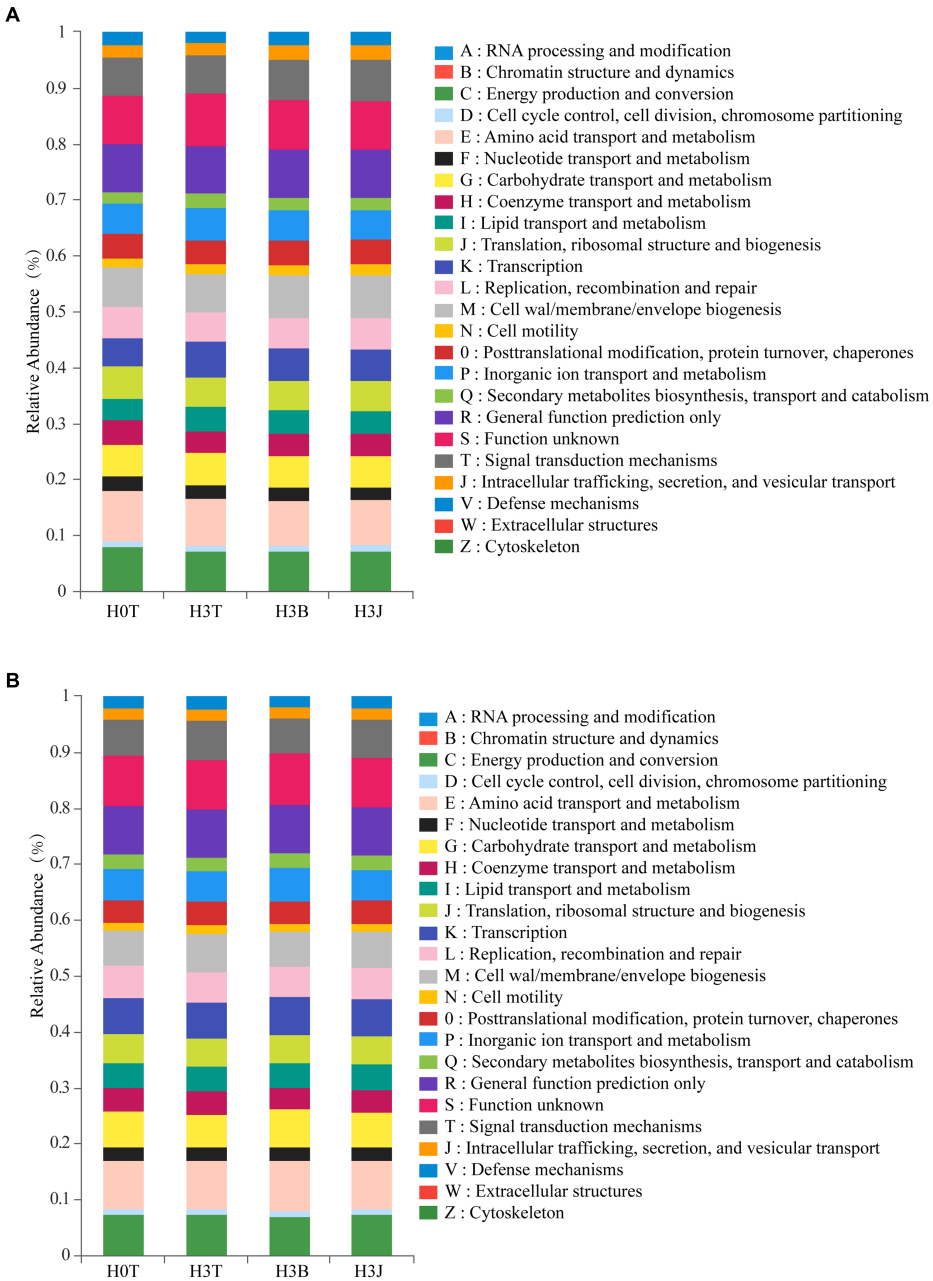
Prediction of soil bacterial functions based on PICRUSt2 in 2019 **(A)** and 2020 **(B)**.

In 2019 (Figure 5A), the Energy production and metabolism, Amino acid transport and metabolism, and General function prediction only were the main soil bacterial functions, and the Amino acid transport and metabolism and General function prediction only in the H3T group were enhanced compared with those in the H0T group, indicating that the addition of exogenous Cd led to the enhancement of the two functions. Besides, the Replication, recombination and repair was also enhanced in the H3B and H3J group compared with that in the H3T group. Similar results were obtained in 2020 (Figure 5B).

### 3.6 Correlation analysis between dominant species and soil/cotton Cd content

**Figure 6:**
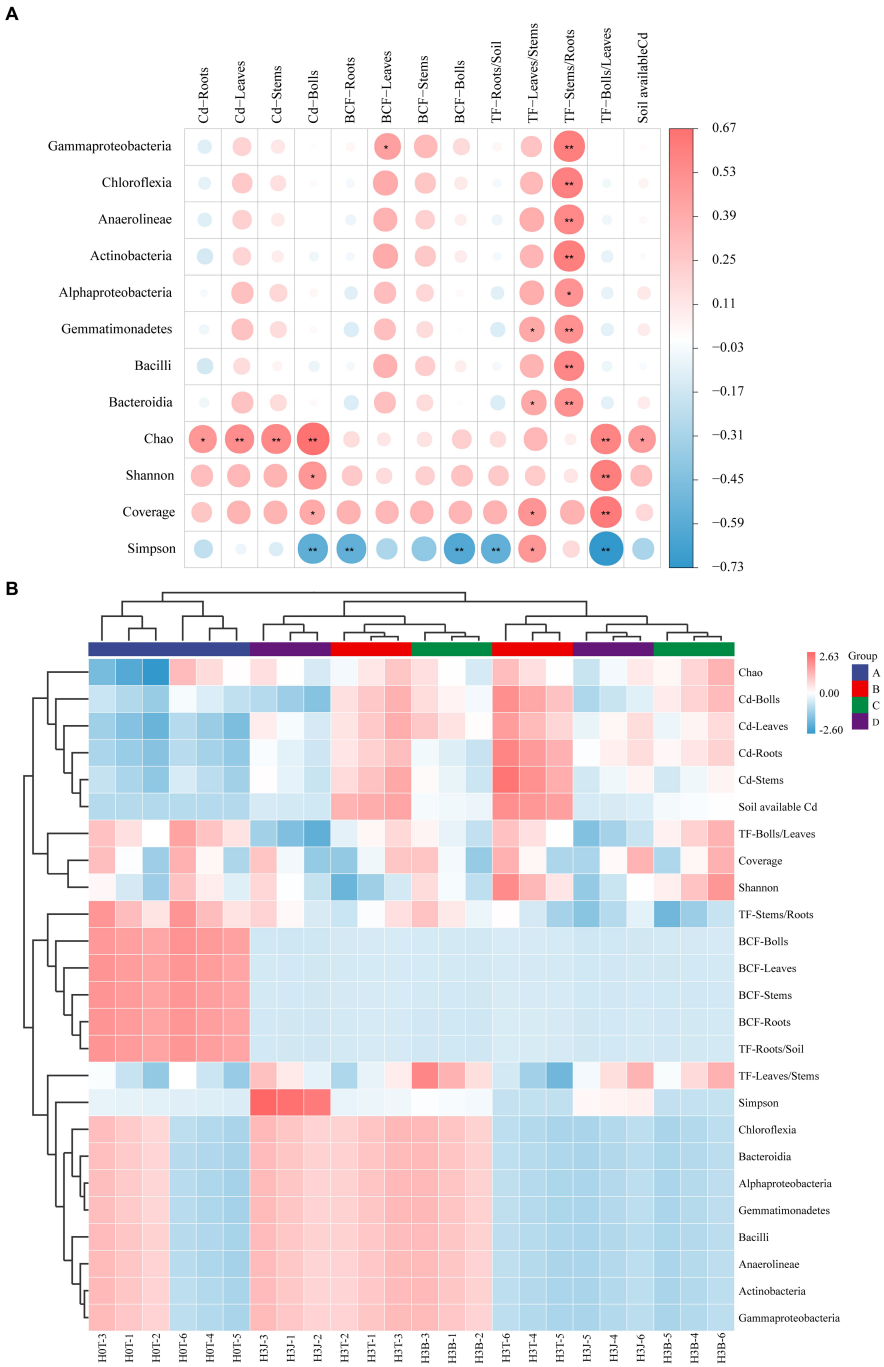
Correlation analysis between soil/cotton Cd enrichment and transport and soil bacterial community. (A) Correlation analysis; (B) Heatmap analysis.

The heatmap showed that TF-Leaves/Stems was positively correlated with the abundance of *Gammaproteobacteria*, *Chloroflexia, Anaerolineae*, *Actinobacteria*, *Gemmatimonadetes*, *Bacilli*, and *Bacteroidia* (*p* < 0.01; Figure 6A). TF-Bolls/Leaves was positively correlated with Chao 1, Shannon, and Coverage index (*p* < 0.01), but negatively correlated with Simpson index (*p* < 0.01). Chao 1 index was positively correlated with Cd-Stems, Cd-Leaves, and Cd-Bolls (*p* < 0.01), and Simpson index was negatively correlated with Cd-Bolls, BCF-Roots, BCF-Bolls, TF-Roots/Soil, and TF-Bolls/Leaves (*p* < 0.01; Figure 6A). The bacterial community structure in the H3T, H3B, and H3J groups were similar, and the effects of H3T, H3B, and H3J treatments on BCF-Bolls, BCF-Leaves, BCF-Stems, BCF-Roots, and TF-Roots/Soil were similar (Figure 6B).

## 4 Discussion

In 2020, the average contents of Pb, Cd, Hg, and Cu in Xinjiang’s cotton fields were 1.04, 5.73, 2.22, and 1.14 times the threshold values in the *Standard for Risk Control of Arable Soil Pollution* (GB 15618-2018), respectively, while the average contents of Cr, As, and Ni were lower than the threshold values. Previous study has found that Pb, Cd, As, and Cu pollution hotspots are widely distributed in southwest China, the North China Plain, the Yangtze River Basin, the Yangtze River Delta, and the Pearl River Delta (Shi et al., 2023). Besides, a survey of 341 arable soil samples in Shaanxi Province in northwest China found that both Cd and Pb accumulated in large quantities in the soil, and the contents of Cd, Hg, and Zn increased with the increase of cropping years (Jing et al., 2023). This study results suggest that the heavy metal pollution of arable soil in the study area deserves attentions. Among heavy metal polluted cotton fields (with heavy metal content exceeding the background value in Xinjiang), Cd polluted cotton fields had the highest proportion (88.33%; Table 2). In terms of the coefficient of variation, the variations of soil As, Cd, Cr, Cu, Ni, and Pb were all medium, among which, the coefficient of variation of Cd and Pb were greater than that of other heavy metals. In addition, the dispersion degree of Cd and Pb was high (Table 2), indicating that their contents varied greatly in different cotton fields. This also indicates that the heavy metal content in the study area is affected by random factors such as industrial activities, excessive application of fertilizers, and agricultural irrigation, and Cd is the most abundant heavy metal in the cotton fields of Xinjiang (Table 2; Huang et al., 2021; Yuan et al., 2021).

The TF and BCF are the two main parameters for evaluating the uptake and accumulation of heavy metals in plants. If TF and BCF are greater than 1, it indicates the uptake of heavy metals by plants; If TF and BCF are less than 1, it indicates the exclusion of heavy metals (Ahmad et al., 2017). In this study, the TF and BCF of Cd in cotton roots, stem, leaves, and buds/bolls were less than 1, and the TF and BCF of Cd in roots were greater than those in other organs (Figure 1; Supplementary Figure S2). However, Li et al. (2012) reported that the BCF of the vegetative organs, aerial parts, and whole plants of three cotton varieties were greater than 1. This difference may be due to: 1) differences in cotton varieties lead to different enrichment and transport of Cd in cotton organs; and 2) differences in soil pH lead to different bioavailability and migration of Cd in soil (the soil is weakly alkaline in this study, while the soil is acidic in the study of Li et al. (2012) and Nawab et al. (2016). It has been reported that the TFs and BCFs of heavy metals of corn grains are different under the treatments of biochar derived from different raw materials (For BCF, *Ganoderma lucidum* substrate derived biochar treatment (0.0651) < mushroom substrate derived biochar treatment (0.0817) < *Hericium* substrate derived biochar treatment (0.0742); For TF, mushroom substrate derived biochar treatment (0.204) < *Ganoderma lucidum* substrate derived biochar treatment (0.211) < *Hericium* substrate derived biochar treatment (0.222; Li et al., 2012). In this study, the application of BC and BF increased the transport of Cd from stems to leaves at different Cd levels. This indicates that in addition to cotton roots, cotton leaves also have strong Cd enrichment and transport capacities, which may be related to the bioavailability of Cd in the soil and soil physicochemical properties (Noli and Tsamos, 2016). Studies have shown that the content of Cd in wheat grains is significantly reduced by 26.13%–46.43% compared with the control after the application of 1.25% rice straw biochar, vegetable peel biochar, corn straw biochar, and rice husk biochar, and the TF and BCF of Cd are also significantly reduced (Song J. W. et al., 2022; Amin et al., 2023; Sun T. et al., 2023). In this study, the application of BC and BF significantly reduced the content of available Cd in soil (Supplementary Table S2), the absorption of Cd by various organs of cotton (Supplementary Table S3), the BCF of Cd in cotton (Figures 1A–D; Supplementary Figures S2A,B), and increased the transport of Cd from roots to stems (1, 2, 4 mg·kg^−1^ Cd levels). The difference in TF may be due to the different raw materials of biochar, the different heavy metal absorption capacity and the different heavy metal tolerance in different crops (Amin et al., 2023).

The application of heavy metal-tolerant bacteria and biofertilizer can reduce the bioavailability of soil heavy metals and reduce the absorption of heavy metals by plants. Abdelkrim et al. (2020) showed that the total heavy metal content of the soil treated with heavy metal-tolerant bacteria (PGPR) was less than that of the soil without PGPR. These scholars provided the following reasons: (1) the PGPR converted Cd and Pb into bioavailable forms in the rhizosphere, which enhanced the absorption of heavy metals by alfalfa. (2) The PGPR released degrading enzymes, organic acids, and metal chelates (such as siderophores) into the rhizosphere, enhancing heavy metal uptake and accumulation in alfalfa roots (Abdelkrim et al., 2020). This study obtained similar results, that is, the application of BF reduced the content of available Cd in soil and the content of Cd in cotton organs (Figure 1; Supplementary Figure S2; Supplementary Tables S2, S3). This may be due to the abundant organic matter in BF, as well as differences in bacterial species and their tolerances (Sahar et al., 2019). In this study, the effects of BC and BF on soil available Cd content and cotton Cd enrichment and transport were inconsistent (Figure 1; Supplementary Figure S2; Supplementary Tables S2, S3), and the performance of BF in reducing cotton BCF was superior to that of BC (Figures 1A,B). This may be related to the differences in soil bacterial community composition and metabolites caused by the application of BC and BF and the differences in the physical and chemical properties of BC and BF (Ma et al., 2022; Zhu Y. Q. et al., 2022). In addition, the application of BC and BF increased the abundance of Amino acid transport and metabolism and Replication, recombination and repair metabolism-related bacteria in the soil. This promotes cotton growth and improves cotton resistance to heavy metal stress (Hasnain et al., 2023).

Bacteria have mechanisms that resist heavy metal stresses, such as biological detoxification, efflux, and cellular resistance to oxidative stress. Besides, bacteria also play a key role in the redox reactions, methylation, and demethylation of heavy metals, and the formation of organometallic complexes (Kou et al., 2023). Wang Z. et al. (2020) found that *Actinobacteria* and *Chloroflexia* were the dominant species in Cd-polluted soils, and soil Cd and Pb contents were positively correlated with the abundance of *Actinomarinales* (*p* < 0.001), *Pedomicrobium* (*p* < 0.05), *Xanthobacteraceae* (*p* < 0.001), and *Alphaproteobacteria* (*p* < 0.001; Kou et al., 2023). Sun Y. et al. (2023) reported that after applying rice husk powder derived biochar into Cd-polluted soil, the relative abundance of *Proteobacteria*, *Acidobacteria*, *Bacteroidetes*, *Gemmatimonadetes*, *Actinobacteria*, *Planctomycetes* and *Chloroflexi* were increased by 28.69%–33.36%, 17.94%–20.19%, 7.10%–9.09%, 9.06%–11.69%, 5.02%–6.98%, 3.32%–6.14% and 2.69%–5.28%. Jin et al. (2021) reported that *Proteobacteria*, *Chloroflexi*, *Acidobacteria*, *Actinobacteria*, *Bacteroidetes*, and *Planctomycetes* were the dominant species in the soil after the application of biofertilizer. It can be seen that the changes in soil microbial community structure in previous studies are different from those in this study. This may be due to differences in soil pH, biochar and biofertilizer dosages, and agricultural managements (such as fertilizer application rates and irrigation volumes; Sun T. et al., 2023). In this study, in the 2 years, *Alphaproteobacteria*, *Gammaproteobacteria*, *Blastocatellia*, and *Gemmatimonadetes* were the dominant species under BC and BF treatments (Figure 4). This indicates that the above taxa have a high resistance to Cd stress. Study has shown that long-term heavy metal pollution leads to changes in soil microbial community structure and increases in the relative abundance of heavy metal-tolerant microorganisms and soil microbial diversity (Han et al., 2020). This may be due to that heavy metal-resistant microorganisms have multiple heavy metal oxidase genes involved in heavy metal fixation and resistance. These microorganisms participate in nitrogen nitrification and denitrification, which provides nitrogen for plants and improves the living environment of microorganisms (Han et al., 2020; Cheng et al., 2023). In addition, the pore-rich structure and abundant carbon and nitrogen of BC provide favorable conditions for the growth and reproduction of bacteria, and the large number of bacteria and rich nutrients in BF can increase the diversity of soil bacteria that fix and adsorb soil heavy metals (Figures 2, 3; Ahamad et al., 2023; Wei et al., 2023; Yu et al., 2023). It was also found that there was a positive correlation between soil dominant bacteria and TF-Stems/Roots (*p* < 0.05). This indicates that the dominant species under BC and BF treatments promote the transport of Cd from cotton roots to stems (Figure 6A).

This study found that *Alphaproteobacteria*, *Gemmatimonadetes*, *Bacilli*, and *Vicinamibacteria* were differential species in H3B *vs* H3T. Many studies have shown that *Alphaproteobacteria*, *Gemmatimonadetes*, and *Bacilli* are the dominant species under heavy metal stress conditions. These species play an important role in resisting exogenous Cd stress and reducing the toxicity of heavy metals to plants (Czarny et al., 2020; Qi et al., 2022; Sun et al., 2022). *Gemmatimonadetes* can indicate heavy metal pollution in arable soil as their relative abundance increases significantly with the increase of pollutant concentration in the soil (Niepceron et al., 2013). *Vicinamibacteria* abundance is negatively correlated with soil available Cd content and positively correlated with soil total phosphorus content. The anionic groups on *Vicinamibacteria* cell wall such as hydroxyl, carboxyl, amino, and amide groups can bind with heavy metals to reduce their bioavailability (Yu et al., 2023). This study found that BC and BF treatments had a certain effect on the diversity, relative abundance, and function of soil bacterial communities, which ultimately improved the quality of Cd-polluted soil. This further confirms that the diversity, function, and relative abundance of soil bacteria could be used as evaluation indicators for soil quality (Tang et al., 2019). It was worth noting that BF treatment had a better effect on reducing the enrichment of Cd in cotton than BC treatment.

## 5 Conclusion

In this study, Cd was the most abundant heavy metal in the cotton fields of Xinjiang. Both biochar and biofertilizer could reduce the transport of Cd from soil to roots and the enrichment of Cd in cotton organs. However, biofertilizer was superior to biochar in reducing Cd transport and enrichment. The dominant bacteria in soil (*Gammaproteobacteria*, *Chloroflexia*, *Anaerolineae*, *Actinobacteria*, *Alphaproteobacteria*, *Gemmatimonadetes*, *Bacilli*, and *Bacteroidia*) were significantly associated with the transport of Cd from cotton roots to stems under biochar and biofertilizer treatments. Besides, biochar and biofertilizer treatments increased soil bacterial diversity, and reduced the transport of Cd from soil to the aboveground organs of cotton. Biochar treatment mainly increased the relative abundance of *Gemmatimonadetes*, which in turn reduced the bioavailability of soil Cd and the enrichment of Cd in various organs of cotton. Biofertilizer treatment mainly regulated the abundance of *Alphaproteobacteria*, *Gemmatimonadetes*, *Bacilli*, and *Vicinamibacteria* to enhance the metabolic function Replication, recombination and repair, to reduce the enrichment of Cd in cotton. This study deepens our understanding of the remediation of soil Cd pollution by BC and BF from the perspective of soil microbial community, and provides a reference for the selection of soil heavy metal pollution remediation technique in Xinjiang, China.

## Data availability statement

The datasets presented in this study can be found in online repositories. The names of the repository/repositories and accession number(s) can be found in the article/Supplementary material.

## Author contributions

YZ: Conceptualization, Data curation, Formal asnalysis, Funding acquisition, Investigation, Methodology, Writing – original draft, Writing – review & editing. MA: Writing – review & editing. TA: Writing – review & editing. HW: Resources, Validation, Writing – review & editing.
