# Supplementary material for: Differences in soil bacterial community structure during the remediation of Cd-polluted cotton fields by biochar and biofertilizer in Xinjiang, China
Source: Front Microbiol. 2024 Feb 9;15:1288526. doi: 10.3389/fmicb.2024.1288526 (PMC10884324; doi:10.3389/fmicb.2024.1288526)
Supplement: Supplementary file 1 [file Data_Sheet_1.docx]

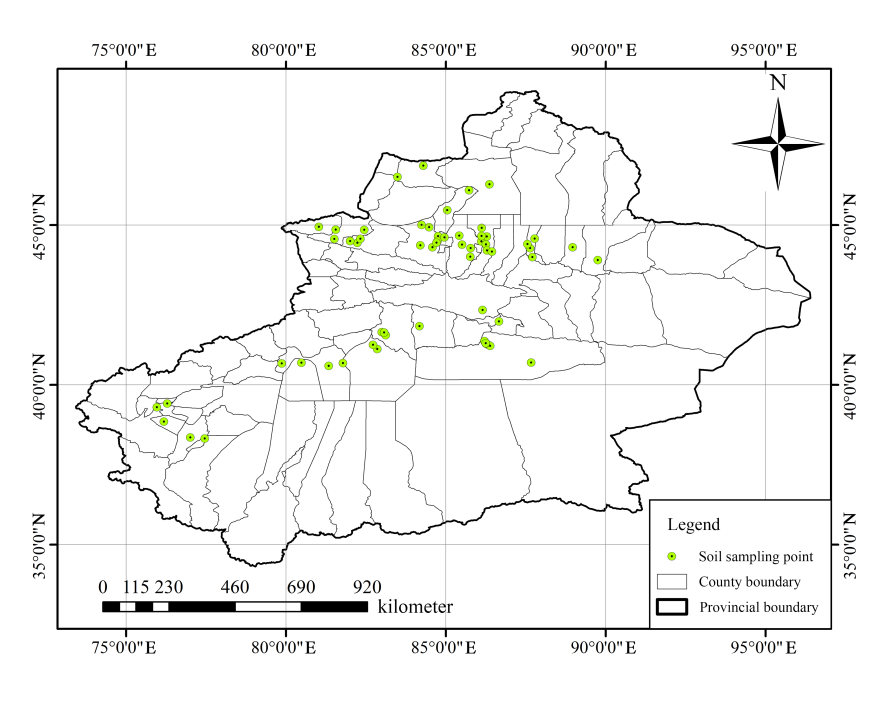


**Figure S1** Distribution of cotton fields for soil sampling


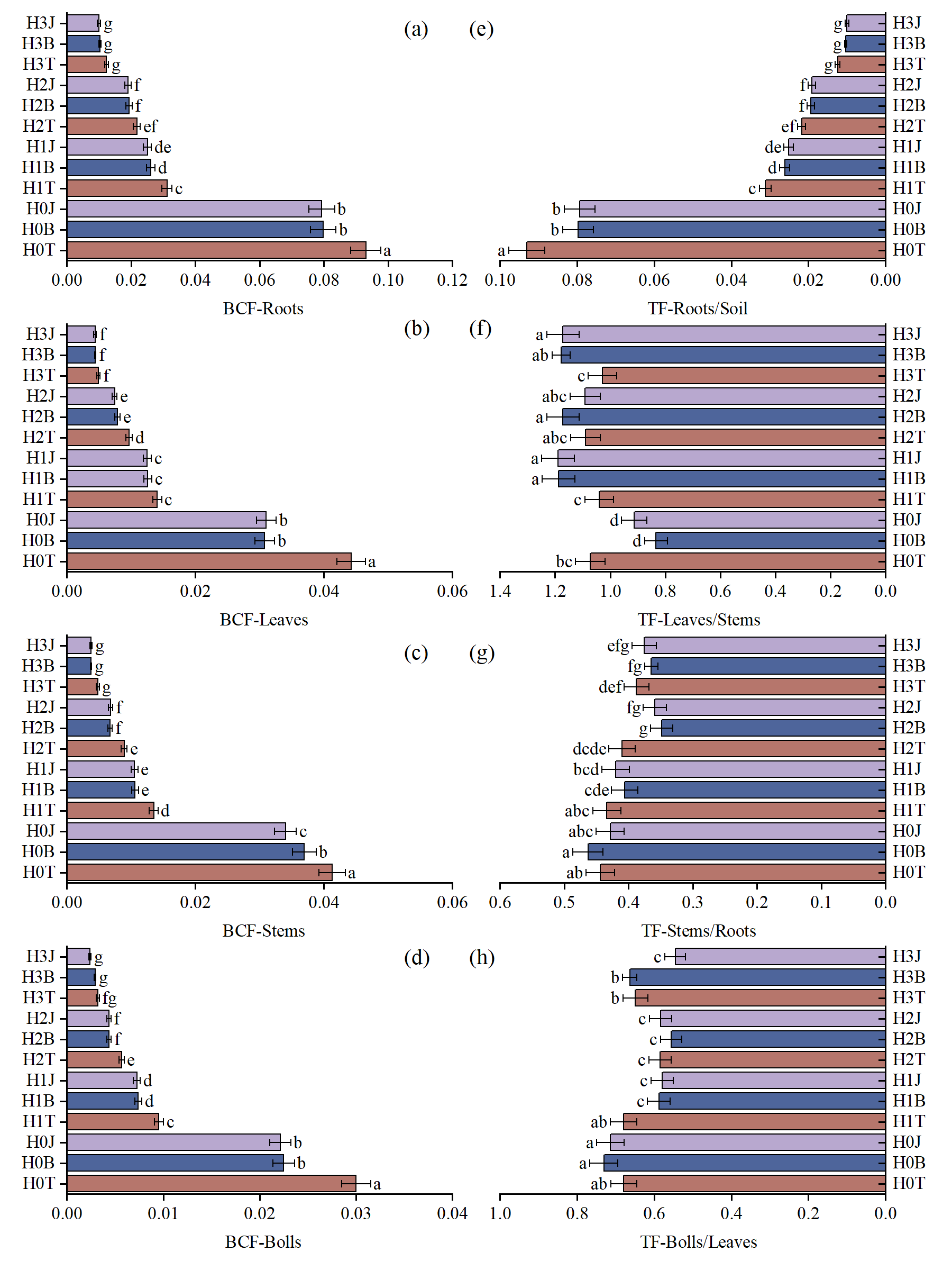


**Figure S2** Bioaccumulation coefficients (BCFs) and Translocation factor (TF) of Cd in cotton organs (2020). Different lowercase letters indicate significant difference between groups at *p* < 0.05. Notes: T, no modifiers; B, 3% biochar was applied; J, 1.5 % biofertilizer was applied; H0, no Cd; H1, 1 mg·kg^-1^ of Cd was applied; H2, 2 mg·kg^-1^ of Cd was applied; H3, 4 mg·kg^-1^ of Cd was applied. The same below.

**
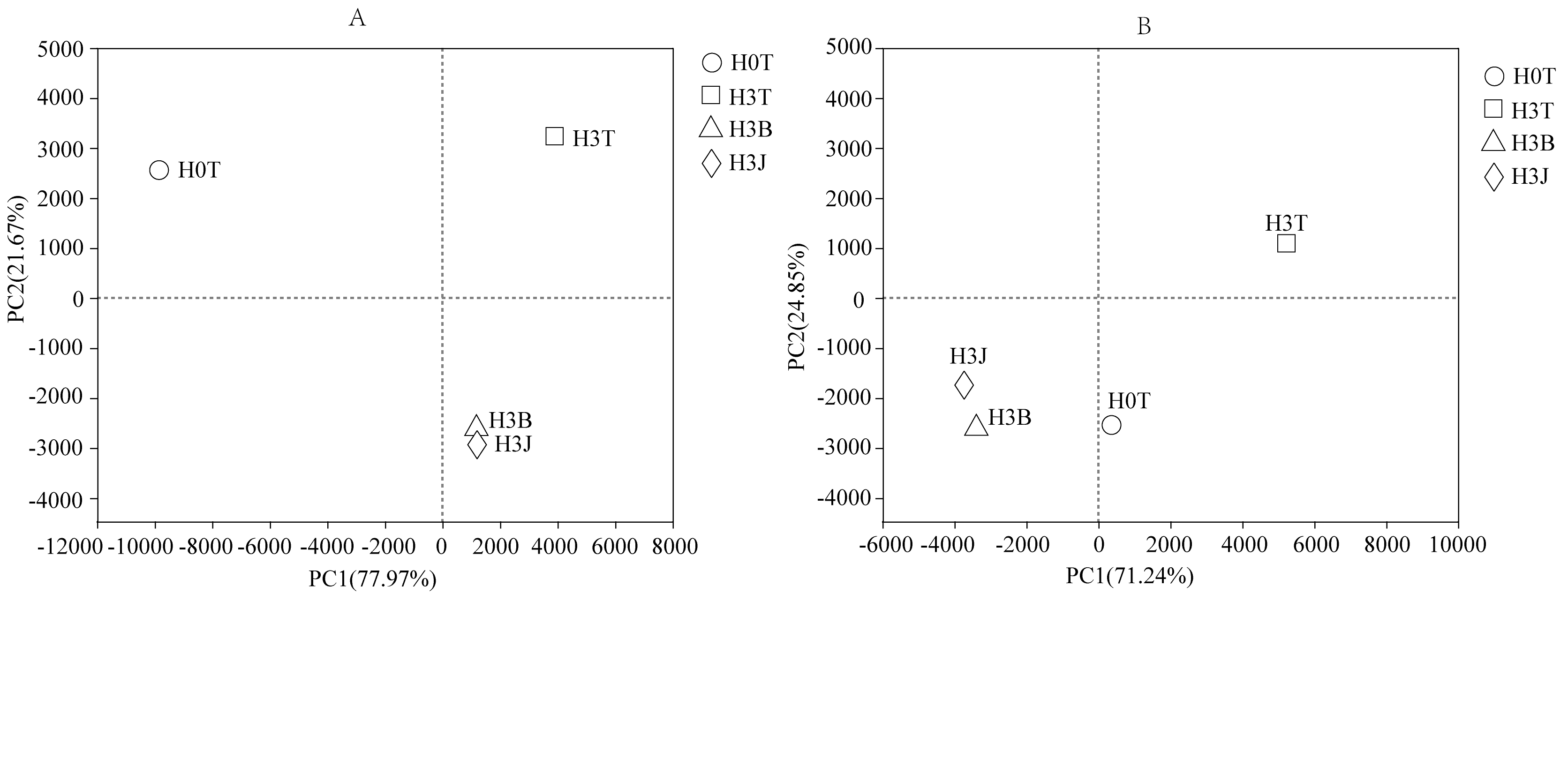
**

**Figure S3** Effect of biochar (B) and biofertilizer (J) on the microbial β-diversity in Cd contaminated soil. A, 2019; B, 2020.


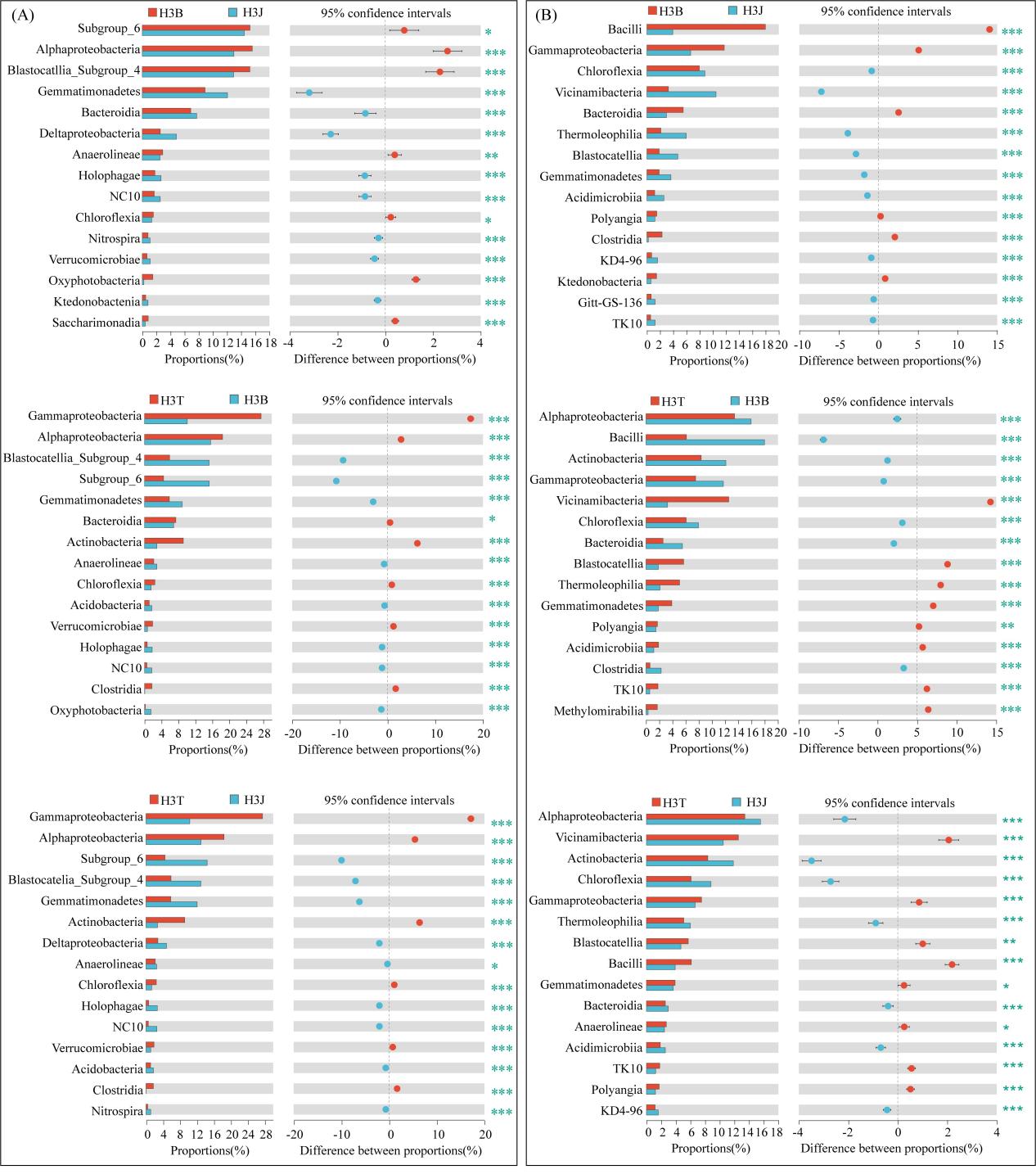


**Figure S4** Differences in soil microbial community structure between groups in 2019 (A) and 2020 (B)

**Table S1** Physicochemical properties of biochar, biofertilizer, and soil

| Property | Cotton straw-derived biochar | Compound *Bacillus* fertilizer | Soil |
| --- | --- | --- | --- |
| pH | 9.50 | 7.8 | 7.76 |
| Total nitrogen (g·kg^-1^) | 0.89 | 91 | 0.46 |
| Total phosphorus (g·kg^-1^) | 2.54 | 62.2 | 0.82 |
| Organic matter (g·kg^-1^) | 625 | 422 | 14.73 |
| Total potassium (g·kg^-1^) | 8.62 | 86.1 | 246.83 |
| Total cadmium (mg·kg^-1^) | 0.002 | 0.0001 | 0.25 |
| Available cadmium (mg·kg^-1^) | - | - | 0.121 |
| Salinity (g·kg^-1^) | - | - | 3.36 |
| Carboxyl (mmol·g^-1^) | 0.20 | - | - |
| Lactone (mmol·g^-1^) | 0.25 | - | - |
| Phenolic hydroxyl (mmol·g^-1^) | 0.21 | - | - |
| The Colony-Forming Units | - | >20 billion·g^-1^ | - |

**Table S2** Effects of amendments on soil available Cd (mg · kg^-1^)

| Treatments | 2019 Year-120 DAS | 2020 Year-120 DAS |
| --- | --- | --- |
| H0T | 0.1261 d | 0.1294 e |
| H0B | 0.0148 gh | 0.0152 g |
| H0J | 0.0051 i | 0.00526 g |
| H1T | 0.185 d | 0.1995 d |
| H1B | 0.0882 ij | 0.0938 f |
| H1J | 0.0582 k | 0.0621 f |
| H2T | 0.50474 d | 0.5675 b |
| H2B | 0.32154 g | 0.349 c |
| H2J | 0.17578 i | 0.1897 d |
| H3T | 1.132 b | 1.2914 a |
| H3B | 0.4501 de | 0.4967 b |
| H3J | 0.2905 h | 0.3157 c |

Notes: T, no modifiers; B, 3% biochar was applied; J, 1.5 % biofertilizer was applied; H0, no Cd; H1, 1 mg·kg^-1^ of Cd was applied; H2, 2 mg·kg^-1^ of Cd was applied; H3, 4 mg·kg^-1^ of Cd was applied. Different lowercase letters indicate significant differences between groups (ANOVA, Tukey's test, *p* < 0.05). The same below.

**Table S3** Effects of amendments on Cd content in various organs of cotton (mg · kg^-1^)

| Treatments | 2019 | | | | 2020 | | | |
| --- | --- | --- | --- | --- | --- | --- | --- | --- |
|  | Root | Leaf | Steam | Boll | Root | Leaf | Steam | Boll |
| H0T | 0.0336  d | 0.0159  de | 0.0149cd | 0.0103  c | 0.0347  de | 0.0165  e | 0.0154d | 0.0112  c |
| H0B | 0.0267  e | 0.0109  f | 0.0089h | 0.0075  e | 0.0298  e | 0.0115  f | 0.0138  ef | 0.0084  f |
| H0J | 0.0256  e | 0.0109  f | 0.0109g | 0.0087  d | 0.0294  e | 0.0115  f | 0.0126  f | 0.0082  f |
| H1T | 0.0373  cd | 0.017  cd | 0.0159bc | 0.0106  c | 0.0408  bc | 0.0184  cd | 0.0177  e | 0.0125  b |
| H1B | 0.0325  d | 0.0159  de | 0.0122  f | 0.0078  e | 0.0342  de | 0.0165  e | 0.0139  ef | 0.0097  e |
| H1J | 0.0326  d | 0.015  e | 0.0123  f | 0.005  f | 0.0329  e | 0.0164  e | 0.0138  ef | 0.0095  e |
| H2T | 0.0435  b | 0.0177  c | 0.0192a | 0.0119  b | 0.0491  b | 0.0219  a | 0.0201b | 0.0128  b |
| H2B | 0.0359  bc | 0.0172  cd | 0.0132ef | 0.009  d | 0.0436  bc | 0.0178de | 0.0152de | 0.0099  de |
| H2J | 0.0366  bc | 0.0162  cde | 0.0138de | 0.0099  c | 0.0429  cd | 0.0168  e | 0.0154d | 0.0098  e |
| H3T | 0.0488  a | 0.0216  a | 0.0197a | 0.0135  a | 0.0557  a | 0.0222  a | 0.0216  a | 0.0144  a |
| H3B | 0.0399  bc | 0.0206  ab | 0.0167b | 0.0123  b | 0.0463  bc | 0.0199bc | 0.0169  c | 0.0132  b |
| H3J | 0.0405  b | 0.0192  b | 0.0166b | 0.0099  c | 0.0451  bc | 0.0198bc | 0.0169  c | 0.0108  cd |
